# Supplementary material for: Bidirectional generation of structure and properties through a single molecular foundation model
Source: Nat Commun. 2024 Mar 14;15:2323. doi: 10.1038/s41467-024-46440-3 (PMC10940637; doi:10.1038/s41467-024-46440-3)
Supplement: Supplementary file 1 — Supplementary Information [file 41467_2024_46440_MOESM1_ESM.pdf]

Supplementary Information

	model	Validity(V) in %	Uniqueness(U) in %	Novelty(N) in %	V× U× N in %
unconditonal	SMILES RNN[2]	93.2	-	89.9	83.8
	SMILES VAE[2]	80.4	-	79.3	63.8
	GraphVAE-NoGM[3]	81.0	24.1	61.0	11.9
	molGAN[4]	98.1	10.4	94.2	9.61
	$\text{SPMM}_{unconditional}{}^{*}$	97.1	99.1	95.0	91.4
conditional	scaffold-GGM†[5]	96.5	85.6	99.0	81.8
	$\text{SPMM}_{deterministic}{}^{*}$	99.5	99.9	96.1	95.5
	$\text{SPMM}_{stochastic}{}^{*}$	99.3	99.9	98.4	97.6

Supplementary Table 1: **Validity, uniqueness, novelty, and their accumulated product of the molecules generated by the Structure-Property Multi-Modal foundation model (SPMM) and other conditional/unconditional molecular generative models. The benchmark results were taken from molGAN[4] and scaffold-GGM[5]. †The metrics are the mean of three separate models trained for different single-property conditions, namely octanol-water partition coefficient (logP), Molar mass, and Topological Polar Surface Area (TPSA). * The SMILES generation was done with 1,000 unseen PVs, with all 53 properties are controlled for conditional generation.**

property	top 15 SMILES tokens with highest attention scores, starting from the highest
NumHAcceptors	[N+](=O)[O-]), C(=O)N1, N2, NC(=O)N, CCCO, N1, [N+](=O)[O-], [nH], c(C(=O)N, (CC(=O)N, CCN(C(=O), CCCCCCCC, c1ccccc1), S(=O)(=O)N, C(=O)N, ...
NumHDonors	Br, (F)(F), C[NH+], CC[NH+], C(F)(F), (F)(F)F), CCCCCCCC, [N+](=O)[O-]), I, NC(=O)N, c(F), C(=O)N, [NH+], (C(=O)N, C(F), ...
ExactMolWt	CCCCCCCC, (F)(F)F), C(F)(F), C(=O)N1, [n, S(=O)(=O), CCN(C(=O), (C(=O)N, CCCCC, I, Br), #, S(=O)(=O)N, O=C(N, N2, ...
RingCount	#, nn, #N), c2c1, =, [N, c(=O), S(=O), (N), (C(=O)N, =N, c2ccc3, [n, c3ccccc3), c1ccc2c(c1), ...
NumAromaticRings	nn, n, cn, nc(, nc1, cn1, c3ccccc3, COC(=O), ncc, c2c1, n1, Cn1, c2ccccc2, =N,)cc2), ...
NumRotatableBonds	CCN(C(=O), CCCCCCCC, C(=O)N1, N1, Cc1ccc(, NC(=O)N, N2, S(=O)(=O)N, c1ccccc1), c3ccccc3, c1ccc2c(, c1cccc(C, CCCCC, CCC1, CCCCC1, ...
TPSA	Br), I, C(=O)[O-], =S, [NH+], Br, CCCCCCCC, C(F)(F), [n, (F)(F)F), CCCCC, C(=O)N1,)N1, [N+](=O)[O-]), [N+](=O)[O-], ...
HeavyAtomCount	Br, I, CCCCCCCC, Br), C(F)(F), (F)(F)F), C(=O)N1, CCCCC, CCN(C(=O), [N+](=O)[O-]), S(=O)(=O), [S, c1ccccc1),)N1, c2ccccc2), ...
MolLogP	[S, (N), CCCCCCCC, s1, (F)(F)F), c(F), C(F)(F), F)cc1, Cn1, c3ccccc, NC(=O)N, Br, c1ccccc1), CCCCC, I, ...
MolMR	I, [N+](=O)[O-], Br), CCCCCCCC, Br, (F)(F)F), CCCCC, [N+](=O)[O-]), C(F)(F), CCN(C(=O), CC(C)(C), [S, NC(=O)N, c(F), (C)C), ...
NOCount	e, N), I, [N+](=O)[O-]), [N+](=O)[O-], O), C(=O)N, CCCCCCCC, c1ccccc1), NC(=O)N, (N), 2)cc1, =[NH+], (F)(F), NC(=O), ...
QED	ccccc, Cc1ccccc1, (Cl), 2)cc1,)cc2), c1ccc2c(c1), Br, c1cccc, Br), c2ccccc2), cs, (F)(F)F), c1ccccc1,)ccc1, c1ccccc1), ...

Supplementary Table 2: **Top 15 SMILES tokens that showed the highest cross-attention score for each property. Only 12 out of 53 properties, that we used in the main manuscript, are listed.**

PV-to-SMILES generation task						
model	sampling	input PV	Validity	Uniqueness	Novelty	normalized RMSE
SPMM (NPP)	deterministic	1,000 unseen PubChem SMILES' PV	0.995±0.001	0.999±0.001	0.961±0.005	0.216±0.004
	stochastic	full PV of the molecule **1**	0.974±0.005	0.905±0.007	0.998±0.003	0.185±0.004
		Molecular weight=150	0.974±0.007	0.945±0.006	0.872±0.007	0.192±0.010
SPMM (MPM)	deterministic	1,000 unseen PubChem SMILES' PV	0.967±0.003	0.990±0.002	0.897±0.009	0.577±0.015
	stochastic	full PV of the molecule **1**	0.956±0.006	0.999±0.001	0.987±0.003	0.625±0.021
		Molecular weight=150	0.952±0.004	0.987±0.003	0.732±0.008	0.262±0.019

MoleculeNet	regression[RMSE, ↓]					classification[AUROC in %, ↑]			
Dataset	Delaney ESOL	LIPO	Freesolv	BACE	Clearance	BBBP	BACE	Clintox	SIDER
SPMM (NPP)	0.817±0.010	0.681±0.004	1.868±0.041	1.041±0.022	42.607±0.675	75.1±0.9	84.4±0.4	92.7±0.7	66.9±0.9
SPMM (MPM)	0.874±0.007	0.725±0.008	1.897±0.060	1.040±0.010	45.821±0.820	72.6±0.7	83.3±0.5	90.4±1.9	65.7±1.5

DILI classification task	Acc in %[↑]	Selectivity in %[↑]	Specificity in %[↑]	AUROC in %[↑]
SPMM (NPP)	84.4	83.9	84.6	92.6
SPMM (MPM)	83.0	81.9	86.9	91.5

Supplementary Table 3: **The ablation studies of replacing the Structure-Property Multi-Modal foundation model (SPMM)'s pre-training objective of Next Property Prediction (NPP) to masked property modeling (MPM), which aims to predict the properties that are replaced with the special masking token similar to BERT[6].**

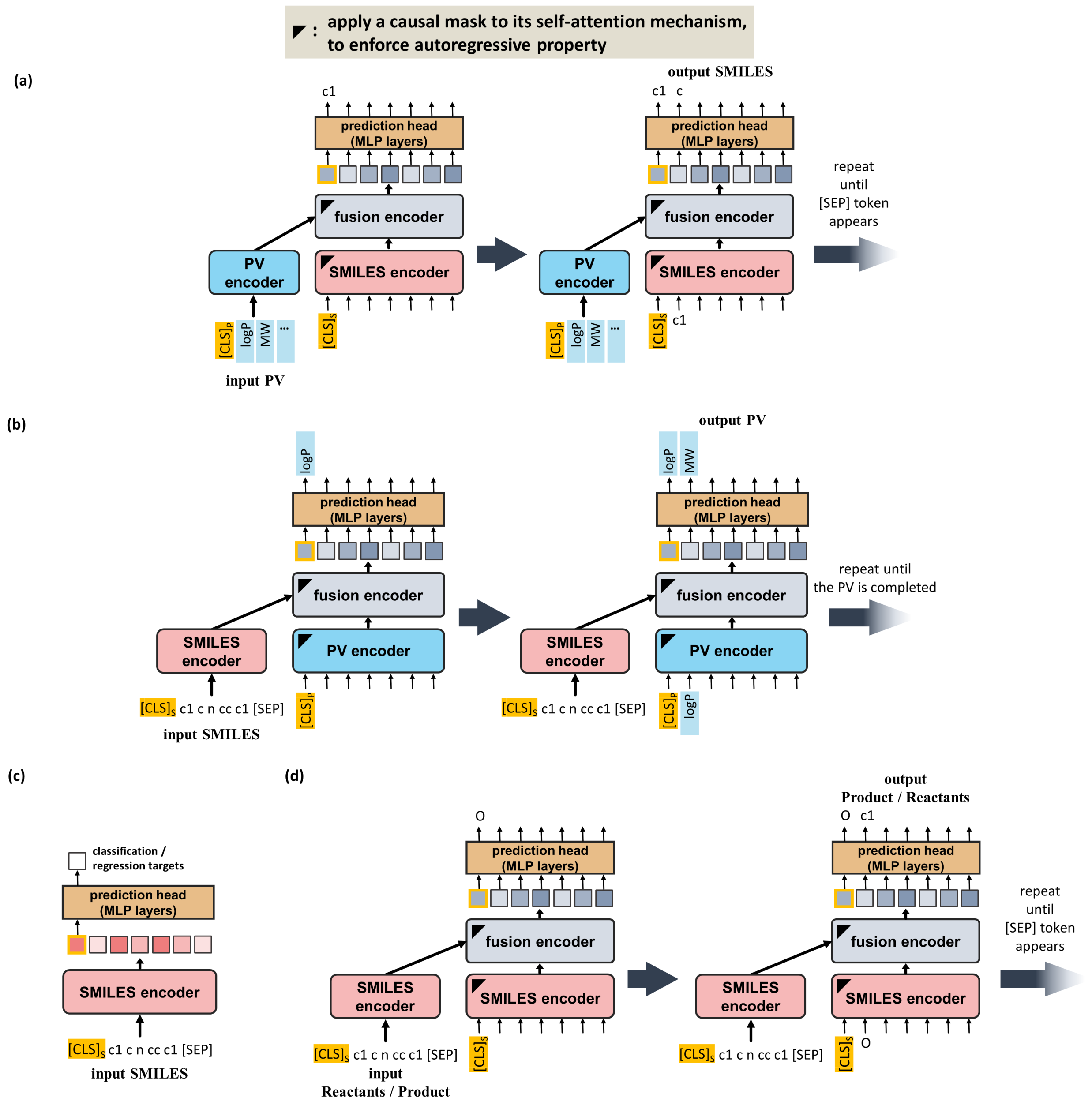

Supplementary Figure 1: **Overview of the inference and fine-tuning of the Structure-Property Multi-Modal foundation model (SPMM) for various downstream tasks: (a) The inference process of pre-trained SPMM for molecule generation. (b) The inference process of pre-trained SPMM for PV generation. (c) The model architecture for MoleculeNet downstream tasks. The SMILES encoder of pre-trained SPMM is used as a backbone. (d) The model architecture for the reaction prediction task. We adopted the SMILES encoder and the fusion encoder of pre-trained SPMM and built a sequence-to-sequence model.**

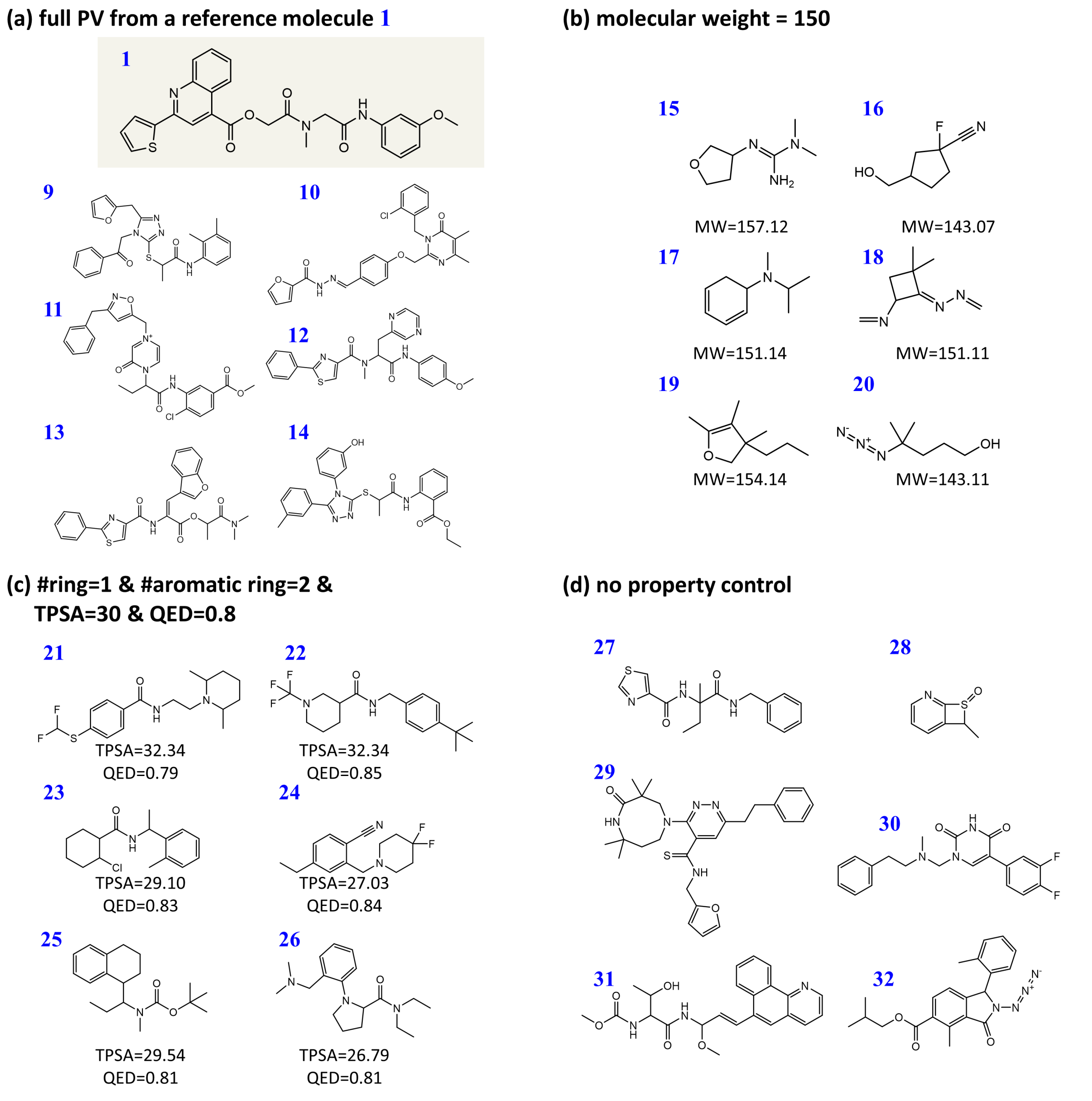

Supplementary Figure 2: **The examples of the output molecules in the stochastic PV-to-SMILES generations by the pre-trained Structure-Property Multi-Modal foundation model (SPMM) with four different given PVs. (a) All 53 properties are controlled with the PV obtained from the molecule 1. (b) Molecular Weight to 150. (c) #ring, #aromatic ring, TPSA, and QED are controlled to 2, 1, 30, and 0.8. (d) Unconditional generation, where every property is replaced with [UNK] token.**

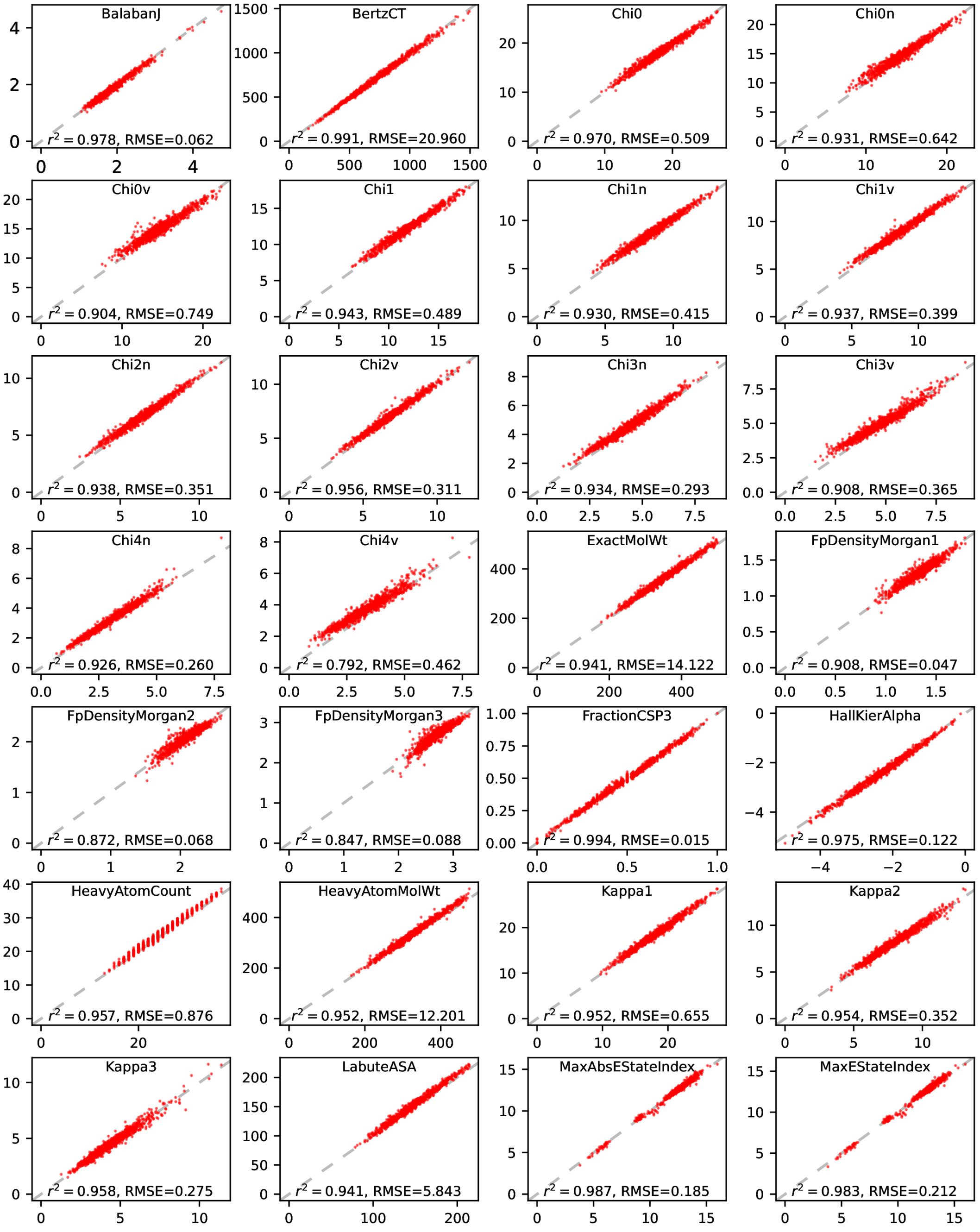

Supplementary Figure 3: **Each property's r^2 score and Root Mean Square Error (RMSE) between the ground truth and the generated Property Vector (PV), for SMILES-to-PV generation with 1,000 unseen ZINC15 molecules. The first 28 out of the total 53 properties are shown in this figure. The names written on the top of each plot are the calculation functions from 'rdkit.Chem.Descriptors' module in RDKit python library[1].**

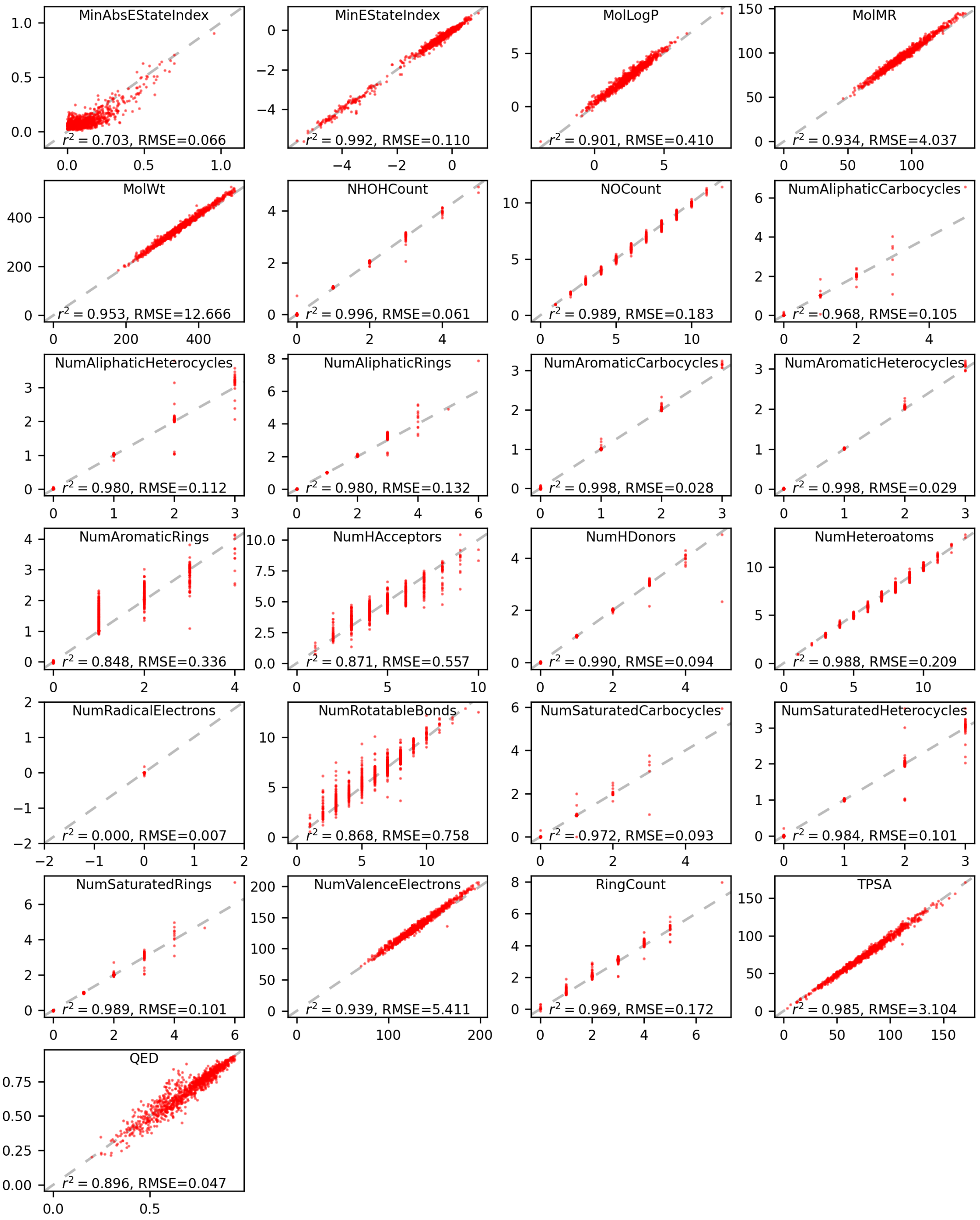

Supplementary Figure 4: **Each property's r^2 score and Root Mean Square Error (RMSE) between the ground truth and the generated Property Vector (PV), for SMILES-to-PV generation with 1,000 unseen ZINC15 molecules. The last 25 out of the total 53 properties are shown in this figure. The names written on the top of each plot are the calculation functions from 'rdkit.Chem.Descriptors' module in RDKit python library[1].**

PV-to-SMILES generation task						
model	sampling	input PV	Validity[↑]	Uniqueness[↑]	Novelty[↑]	normalized RMSE[↓]
Permutation #1	deterministic	1,000 unseen PubChem SMILES' PV	0.995±0.001	0.999±0.001	0.961±0.005	0.216±0.004
	stochastic	full PV of the molecule **1**	0.974±0.005	0.905±0.007	0.998±0.003	0.185±0.004
		Molecular weight=150	0.974±0.007	0.945±0.006	0.872±0.007	0.192±0.010
Permutation #2	deterministic	1,000 unseen PubChem SMILES' PV	0.992±0.002	1.000±0.000	0.991±0.001	0.207±0.003
	stochastic	full PV of the molecule **1**	0.941±0.005	0.909±0.011	0.997±0.01	0.170±0.002
		Molecular weight=150	0.948±0.008	0.989±0.004	0.845±0.015	0.251±0.001
Permutation #3	deterministic	1,000 unseen PubChem SMILES' PV	0.996±0.001	1.000±0.000	0.990±0.002	0.193±0.003
	stochastic	full PV of the molecule **1**	0.960±0.004	0.908±0.006	0.998±0.001	0.167±0.001
		Molecular weight=150	0.968±0.002	0.969±0.003	0.797±0.010	0.188±0.007

SMILES-to-PV generation task	normalized RMSE[↓]	r^2 score[↑]
Permutation #1	0.128	0.923
Permutation #2	0.098	0.939
Permutation #3	0.110	0.925

MoleculeNet	regression[RMSE, ↓]					classification[AUROC in %, ↑]			
Dataset	Delaney ESOL	LIPO	Freesolv	BACE	Clearance	BBBP	BACE	Clintox	SIDER
Permutation #1	0.817±0.010	0.681±0.004	1.868±0.041	1.041±0.022	42.607±0.675	75.1±0.9	84.4±0.4	92.7±0.7	66.9±0.9
Permutation #2	0.834±0.011	0.672±0.008	1.951±0.013	1.042±0.005	42.633±0.438	75.5±0.3	84.4±0.7	92.0±0.5	67.1±0.4
Permutation #3	0.845±0.003	0.681±0.006	1.875±0.017	1.037±0.007	43.754±0.292	75.1±0.2	85.8±0.5	90.7±0.1	67.0±0.8

DILI classification task	Acc in %[↑]	Selectivity in %[↑]	Specificity in %[↑]	AUROC in %[↑]
Permutation #1	84.4	83.9	84.6	92.6
Permutation #2	81.5	79.6	87.7	91.1
Permutation #3	84.2	83.5	80.0	91.6

Reaction prediction tasks	forward acc.[↑]				retro acc.[↑]		
	top-1	top-2	top-3	top-5	top-1	top-5	top-10
Permutation #1	0.915	0.935	0.946	0.954	0.534	0.676	0.703
Permutation #2	0.913	0.934	0.945	0.952	0.538	0.668	0.699
Permutation #3	0.915	0.934	0.946	0.955	0.525	0.680	0.710

Supplementary Table 4: **The performance comparison of three separately pre-trained Structure-Property Multi-Modal foundation model(SPMM)s that utilized their Property Vector (PV) with different property order. Permutation #1 is a property order of PV that was used for the main results.**

	forward acc.[↑]				retro acc.[↑]		
	top-1	top-2	top-3	top-5	top-1	top-5	top-10
augmentation X	0.879	0.914	0.929	0.938	0.445	0.597	0.626
augmentation O	0.915	0.935	0.946	0.954	0.534	0.676	0.703

Supplementary Table 5: **The ablation study of SMILES augmentation on the Structure-Property Multi-Modal foundation model (SPMM)'s forward and retro-reaction prediction task.**

task	Delaney ESOL	LIPO	Freesolv	BACE(reg.)	BACE(cls.)	Clearance	BBBP	Clintox	SIDER	DILI
optimizer	AdamW, weight decay=0.02									
scheduler	cosine + warmup									
batch size	4	16	4	8	16	4	16	16	4	8
learning rate(min, max)	5e-5, 3e-6	2e-5, 3e-6	5e-5, 3e-6	5e-5, 3e-6	5e-5, 5e-6	5e-5, 3e-6	3e-5, 5e-6	5e-5, 5e-6	5e-5, 5e-6	2e-5, 5e-6
epoch	25	25	50	50	20	25	10	15	10	20

task	forward reaction prediction	retro-reaction prediction
optimizer	AdamW, weight decay=0.02	
scheduler	cosine + warmup	
batch size	16	16
learning rate(min, max)	1e-4, 3e-6	1e-4, 5e-6
epoch	100	300

Supplementary Table 6: **Detailed training hyperparameters for fine-tuning the Structure-Property Multi-Modal foundation model (SPMM) in MoleculeNet downstream tasks, DILI classification task, forward reaction prediction task, and the retro-reaction prediction task.**

References

1. Landrum, G. Rdkit: Open-source cheminformatics software (2016).

2. Li, Y., Zhang, L. & Liu, Z. Multi-objective de novo drug design with conditional graph generative model. *Journal of Cheminformatics* **10** (2018).

3. Simonovsky, M. & Komodakis, N. Graphvae: Towards generation of small graphs using variational autoencoders (2018).

4. De Cao, N. & Kipf, T. Molgan: An implicit generative model for small molecular graphs. *arXiv:1805.11973 [cs, stat]* (2022).

5. Lim, J., Hwang, S.-Y., Moon, S., Kim, S. & Kim, W. Y. Scaffold-based molecular design with a graph generative model. *Chem. Sci.* **11**, 1153–1164 (2020).

6. Devlin, J., Chang, M.-W., Lee, K. & Toutanova, K. Bert: Pre-training of deep bidirectional transformers for language understanding (2018).
